# Natural compounds from botanical drugs targeting mTOR signaling pathway as promising therapeutics for atherosclerosis: A review

**DOI:** 10.3389/fphar.2023.1083875

**Published:** 2023-01-20

**Authors:** Qian Wu, Qianyu Lv, Xiao’an Liu, Xuejiao Ye, Linlin Cao, Manshi Wang, Junjia Li, Yingtian Yang, Lanlan Li, Shihan Wang

**Affiliations:** ^1^ Guang’anmen Hospital, Chinese Academy of Chinese Medical Sciences, Beijing, China; ^2^ Capital University of Medical, Beijing, China; ^3^ Beijing Xicheng District Guangwai Hospital, Beijing, China

**Keywords:** mTOR, herbal medicine, rapamycin, autophagy, mechanism, cell senescence, atherosclerosis

## Abstract

Atherosclerosis (AS) is a chronic inflammatory disease that is a major cause of cardiovascular diseases (CVDs), including coronary artery disease, hypertension, myocardial infarction, and heart failure. Hence, the mechanisms of AS are still being explored. A growing compendium of evidence supports that the activity of the mechanistic/mammalian target of rapamycin (mTOR) is highly correlated with the risk of AS. The mTOR signaling pathway contributes to AS progression by regulating autophagy, cell senescence, immune response, and lipid metabolism. Various botanical drugs and their functional compounds have been found to exert anti- AS effects by modulating the activity of the mTOR signaling pathway. In this review, we summarize the pathogenesis of AS based on the mTOR signaling pathway from the aspects of immune response, autophagy, cell senescence, and lipid metabolism, and comb the recent advances in natural compounds from botanical drugs to inhibit the mTOR signaling pathway and delay AS development. This review will provide a new perspective on the mechanisms and precision treatments of AS.

## 1 Introduction

**FIGURE 1:**
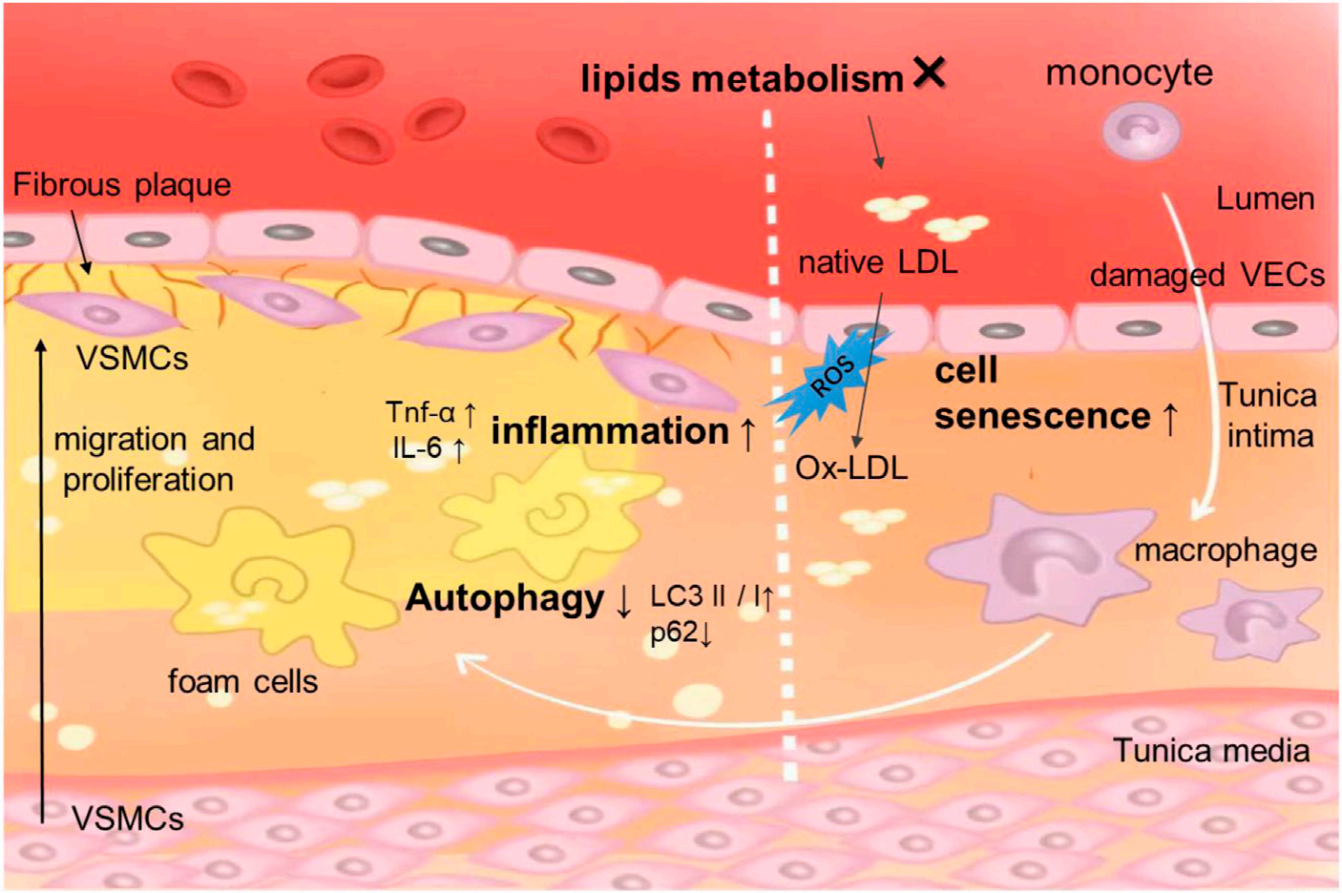
Schematic representation of the pathogenesis of AS. The damage of endothelial cells is the initial lesion of atherosclerosis. Endothelial cell damage that can be caused by disorders of lipid metabolism, monocytes/macrophages, foam cell formation release more inflammatory factors. VECs, VSMCs, macrophages are involved in the process of autophagy. In AS, cell senescence often occurs in VECs, VSMCs.

Atherosclerosis (AS) is a chronic inflammatory disease of blood vessels, which is the main pathologic basis of ischemic cardiovascular diseases, including most cases of myocardial infarction, stroke, and peripheral artery disease (Riggs et al., 2022; Simonetto et al., 2022). Hyperlipidemia, hypertension, and diabetes are major risk factors for AS and are associated with the development, progression, and rupture of atherosclerotic plaque initiation (Yang et al., 2022). Although the therapies for AS have been advanced in recent decades, atherosclerotic cardiovascular disease accounts for most of the mortality worldwide (Libby, 2021a). The pathogenesis underlying AS is complex. Although not fully understood, the occurrence of AS is mainly related to the deposition of inner membrane lipid, endothelial cell injury, adhesion of platelets and leukocytes, invasion and proliferation of smooth muscle cells and collagen fibers, and formation of foam cells (Figure 1) (Bhattacharya et al., 2022; Kim et al., 2022; Lu et al., 2022). Multiple cellular processes and signaling pathways are involved in these biological processes, and the mTOR signaling pathway is one of the regulatory ones (Kong et al., 2022).

The mammalian target of rapamycin (mTOR) is a serine/threonine protein kinase that is evolutionarily highly conserved and can mediate various cellular responses, such as cell growth, metabolism, motility, proliferation, and survival, protein synthesis, cell senescence, apoptosis, and autophagy (Laplante and Sabatini, 2012). Targeting mTOR signaling using specific pharmacological inhibitors may offer a therapeutic promise in inflammatory-associated diseases (Soltani et al., 2018). Recently, much attention has been focused on the potential role of the mTOR signaling pathway as a therapeutic target of atherosclerotic cardiovascular disease. Early studies have shown that the mTOR receptor inhibitor Everolimus firmly inhibits the development of AS in low-density lipoprotein receptor knockout (LDLR^−/−^) mice, providing new insights into the mechanisms of AS (Mueller et al., 2008). Rapamycin is an agent that inhibits the activity of the mTOR signaling pathway. Considering that the current application of Rapamycin is limited by side effects, low biological availability, and lack of targeting, applications of several new technologies targeting atherosclerotic plaques, including 1 and 2, have also been observed in experimental animal models to reduce inflammation and lipid load and shrink plaques, further suggesting that reduced mTOR signaling pathway activity can be used to treat AS. Rapamycin is an inhibitor of the mTOR signaling pathway. By applying advanced technology that makes Rapamycin target atherosclerotic plaques in experimental animals, a reduction in inflammation and lipid load and a shrinking of plaques was also observed in animal models, further suggesting that reduced mTOR signaling pathway activity can promote atheroprotective changes in AS (Huang et al., 2022a; Cheraga et al., 2022; Guo et al., 2022).

For centuries, botanical drugs used in Traditional Chinese Medicine (TCM) have been widely practiced for the prevention and treatment of chronic heart disease. Botanical drugs have attracted widespread attention for their wide range of sources and multi-target effects (Zhi et al., 2023). Various natural agents derived from botanical drugs have been proven to have anti-AS effects (Wang et al., 2019; Penson and Banach, 2021). The current research targeting the mTOR signaling pathway by botanical drugs used in TCM provides new ideas for the treatment of AS (Poznyak et al., 2022). In recent years, an increasing number of studies have been conducted on the mTOR signaling pathway, and the relationship between mTOR and AS has been reviewed in terms of inhibition of inflammatory responses and suppressing the immune response (Cai et al., 2018). However, a review of preclinical evidence for TCM interventions targeting AS mTOR is lacking. Therefore, a comprehensive and systematic clarification of the molecular mechanisms by which natural agents target the mTOR signaling pathway is needed.

**FIGURE 2:**
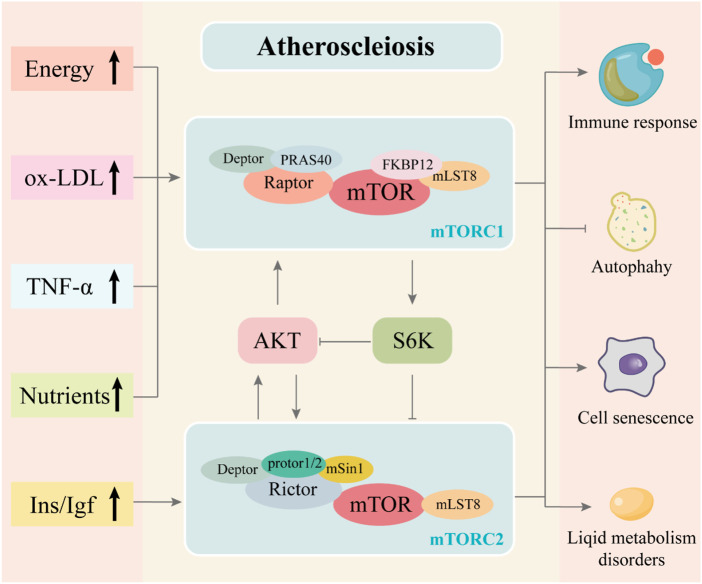
Association of mTOR signaling pathway with AS and the composition of the mTORC1 and mTORC2 complexes. The mTORC1 complex is composed of mTOR, Raptor, mLST8, PRAS40, Deptor and FKBP12. The mTORC2 complex is composed of mTOR, Rictor, mLST8, protor1/2, Deptor and mSin1. The mutual regulation is between mTORC1 and mTORC2. ​ The development of atherosclerosis is often accompanied by abnormal lipid and energy metabolism and increased inflammatory response, which may activate the mTOR signaling pathway through complex mechanisms. These stimuli, as upstream signals of the mTOR signaling pathway, regulate the activity of the mTORC1 and mTORC2 complexes in the mTOR signaling pathway and influence downstream regulation, producing effects that promote immune response and cell senescence, disrupt lipid metabolism and inhibit autophagy, thereby exacerbating atherosclerosis.

Based on the importance of the mTOR signaling pathway in AS, in this review, we first summarized our current understanding of the mTOR signaling pathway’s role in AS from the aspects of immune response, autophagy, cell senescence, and lipid metabolism (Figure 2). Subsequently, we review the preclinical evidence of botanical drugs acting on the mTOR signaling pathway for the prevention and treatment of AS and related pathological processes.

## 2 mTOR signaling pathway participates in AS pathogenesis

The mTOR protein, a member of the phosphatidyl inositol 3-kinase (PI3K) related kinases (PIKKs), is a highly conserved serine/threonine protein kinase that catalyzes the transfer of phosphate to the hydroxyl group of the serine or threonine chains (Liu and Sabatini, 2020). Various proteins interact with mTOR to form two large protein complexes, named mammalian target of rapamycin complex 1 (mTORC1) and mammalian target of rapamycin complex 2 (mTORC2), the core complexes of the mTOR signaling pathway (Yang et al., 2013). More studies on AS currently focus on mTORC1 than mTORC2.

mTORC1 is closely related to autophagy, lipolysis, protein synthesis, and other cell biological processes, which are sensitive to Rapamycin (Ben-Sahra and Manning, 2017). As one of the essential components of mTORC1, the regulatory protein associated with mTOR (Raptor) is a nutrient-sensitive polypeptide bound to mTOR, modulating the kinase activity of mTOR by forming the mTOR-Raptor association, in which the catalytic domain of mTOR phosphorylates substrates only upon binding to Raptor (Kim et al., 2002; Kim and Sabatini, 2004; Avruch et al., 2009). The substrates mainly contain S6 kinase (S6K) and eukaryotic translation initiation factor 4E-binding proteins 1(4E-BP1), which control cap-dependent translation initiation and elongation of mRNA (Ma and Blenis, 2009). Both mTORC1 and mTORC2 compounds contain Mammalian lethal with SEC13 protein 8 (mLST8), an indispensable protein subunit of the mTORC complex that interacts with the mTOR protein kinase domain to stabilize its active site (Saxton and Sabatini, 2017). Unlike Raptor in mTORC1, Rapamycin-insensitive companion of mTOR (Rictor) promotes mTOR substrate recruitment in mTORC2, similar to the effect of another vital component—serum-and glucocorticoid-induced protein kinase 1 (sin1) (Sarbassov et al., 2004; Chen and Sarbassov dos, 2011). Growth factors activate mTORC2; then, mTORC2 phosphorylates substrates, including some AGC-family kinases such as protein kinase B (AKT), serum-and glucocorticoid-induced protein kinase 1(SGK1), and protein kinases C (PKC), subsequently participates in cell survival, regulates ion transport, glucose metabolism, and other activities (Garcia-Martinez and Alessi, 2008; Viana et al., 2018). There is also a mutual regulatory mechanism between mTORC1 and mTORC2. AKT, as well as a negative modulation of the upstream activator of the inhibitory subunit in mTORC1, can be phosphorylated by mTORC2. Meanwhile, S6K, as a substrate of mTORC1, also acts upstream of mTORC2, and after Ins/IGF activation, S6K can inhibit the phosphorylation of IRS1, which in turn suppresses the activation of the PI3K-AKT signaling pathway in the upstream regulation of mTORC2 (Julien et al., 2010). The mTORC complex acts as a hub, coordinating upstream signaling and downstream effects. The mTOR signaling pathway can be activated by growth factors, insulin, ox-LDL, nutrients, and TNF-α, which are also stimuli closely related to the occurrence of AS.

**FIGURE 3:**
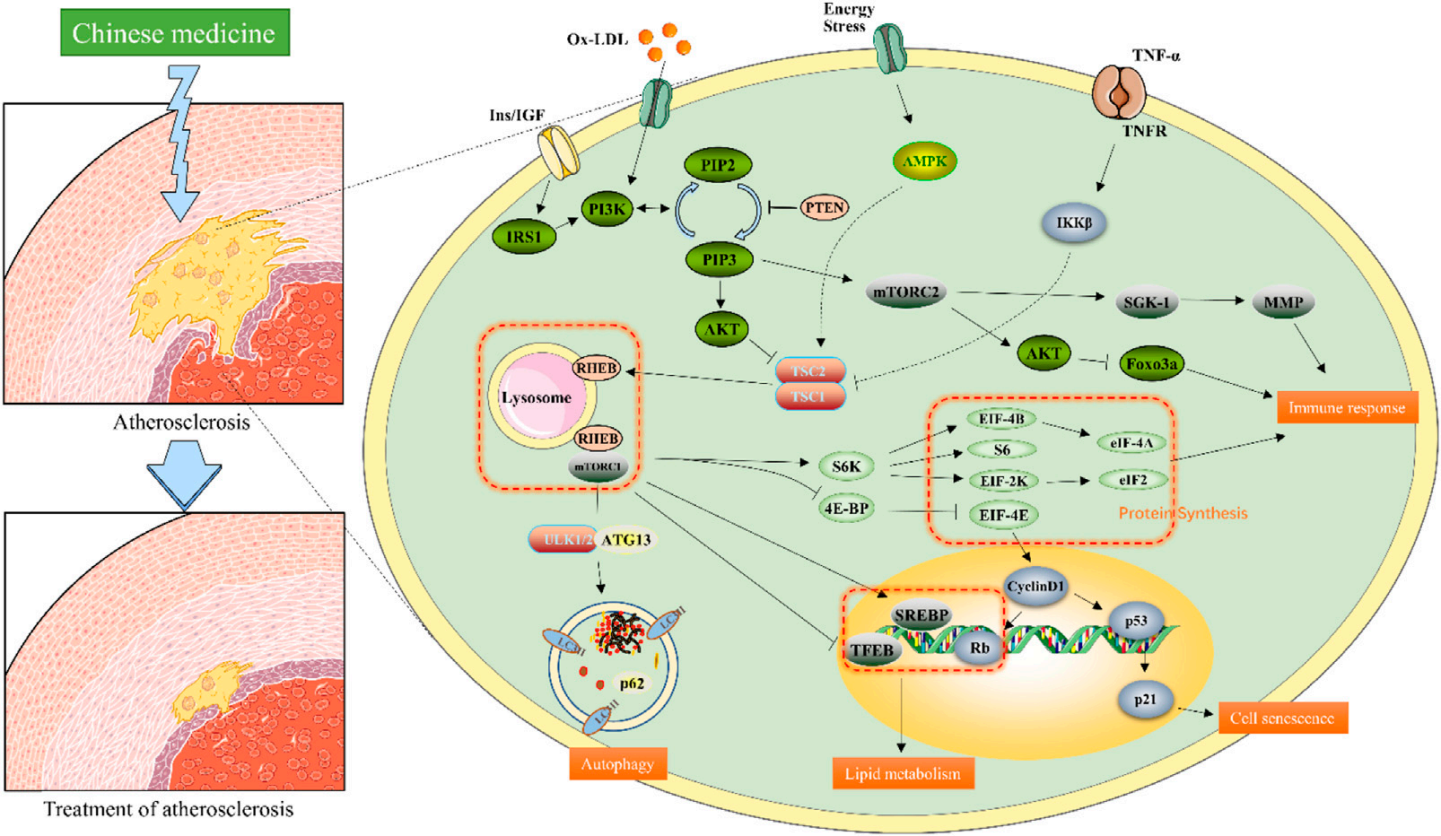
Molecular mechanism of the mTOR signaling pathway associated with AS. When the site of atherosclerosis occurs, TNF-α, lipid deposition, and increased nutrients activate the mTOR signaling pathway. These signal transductions converge on TSC1/2, which in turn inhibits Rheb activity. Rheb regulates mTORC1 activity by modulating conception. mTOR2 is mainly regulated by PI3K/AKT signaling pathway. mTORC1 and mTORC2 work together to regulate downstream proteins. The application of natural compounds from botanical drugs affects the activity of factors related to the mTOR signaling pathway in different ways, thereby increasing autophagy, inhibiting inflammation, delaying aging, and regulating lipid levels.

AS is recognized as associated with immune disorders, but also involves a variety of mechanisms, including the death, autophagy, and senescence of vascular smooth muscle cells (VSMCs) and vascular endothelial cells (VECs), macrophage polarization, death, and autophagy, which are related to the function of mTOR signaling (Jia et al., 2007). The mTOR signaling pathway plays a complex role in the process of AS formation, plaque development, and stabilization. Although the specific mechanism of its intervention remains unclear, studies have shown that immune responses, autophagy, regulation of lipid metabolism disorder, and cell senescence (in which the mTOR signaling pathway is involved) can crucially contribute to AS. Therefore, we will elaborate on the role of the mTOR signaling pathway in AS from the following four aspects; the specific mechanism is shown in Figure 3.

### 2.1 Immune response and mTOR signaling pathway in AS

The immune response is considered a vital component of AS, and the specific mechanism of immune response mediated by the mTOR signaling pathway in AS is complex (Kaldirim et al., 2022). Most studies have focused on drugs that can inhibit mTOR signaling pathway activation, particularly the mTORC1 complex, and the use of Rapamycin was found to significantly reduce the expression of inflammatory factors (Shrimali et al., 2013; Vitiello et al., 2015; Peng et al., 2018). Further study shows that the knockdown of PRAS40, an inhibitory subunit of the mTORC1 complex, promotes TNFα-induced the mTOR signaling pathway, proliferation, upregulation of inflammatory markers, and monocyte recruitment (Aspernig et al., 2019; Dai et al., 2019). The underlying molecular mechanisms are valuable but have not been completely elucidated. The endothelial dysfunction caused by abnormal lipid metabolism, diabetes, and many other factors is the initial stage of AS, which will lead to the adhesion of monocytes and monocyte-derived macrophages to the inner lining of the blood vessels and ingestion of deposited oxidized low-density lipoprotein (ox-LDL), forming foam cells (Hetherington and Totary-Jain, 2022; Scipione and Cybulsky, 2022). Previous studies have shown that ox-LDL upregulated the protein expression of p-mTOR, p-S6K1, p-4EBP1, and the mRNA expression levels of inflammatory cytokines, including interleukin 6 (IL-6), monocyte chemoattractant protein-1 (MCP-1), Toll-like receptor-4 (TLR-4), and tumor necrosis factor-alpha (TNF-α), in THP-1-derived macrophages (Liao et al., 2021). After applying everolimus to inhibit mTORC1, the macrophages in the vascular wall were selectively cleared (Verheye et al., 2007). Furthermore, TNF-α is one of the upstream activators of the mTOR signaling pathway (Gao et al., 2015), which further binds to TNFR on the cell membrane, activates IKKβ, and suppresses the tuberous sclerosis complex 1/2 (TSC1/2) following phosphorylation (Dan and Baldwin, 2008). TSC1/2 is a converging factor for several signaling pathways upstream of the mTOR signaling pathway, including those of PI3K/AKT, MAPK, and AMPK (Sarbassov et al., 2005; Yang et al., 2014; Holczer et al., 2019). TSC1/2 has been shown to regulate the polarization of M1/M2 macrophages (Fang et al., 2015). Notably, it inhibits Ras homolog enriched in the brain (Rheb) protein downstream (Menon et al., 2014). Rheb activates mTORC1 by allosterically realigning the active site residues, putting them into the right register for catalysis, further leading to downstream S6K activation and 4E-BP inhibition (Choo et al., 2008; Yang et al., 2017). Several studies have shown that S6K activation is associated with endothelial cell migration, angiogenesis, T cell activation, and nitric oxide synthase production, and drugs that inhibit the mTOR signaling pathway can protect endothelial cells by reducing S6K activity (Zheng et al., 2007; Habib et al., 2013; Hwang et al., 2015; Jiang et al., 2018; Medina-Jover et al., 2020; Arora et al., 2022).

In another study mTORC2 has also been revealed to be associated with immune responses in AS. Recent research has demonstrated that Rictor-deficient monocytes are unable to effectively move to the sites of inflammation and fully develop into macrophages, reducing the inflammatory response *in vivo* (Jangani et al., 2022). In mice with myeloid lineage-specific Rictor deletion, the survival rate of monocytes/macrophages and mTORC2 activity were significantly decreased (Babaev et al., 2018). Furthermore, SGK-1, the downstream product of mTORC2, regulates metal ion transportation to control cell survival (Garcia-Martinez and Alessi, 2008). SGK1 plays a key role in vascular inflammation during AS due to its participation in the regulation of monocyte/macrophage migration and MMP-9 transcription through the regulation of nuclear factor-κB(NF-κB) (Yamamoto et al., 2022). MMP-9 is an independent predictor of atherosclerotic plaque instability in patients with stable coronary heart disease (Olejarz et al., 2020). Nf-κB-dependent upregulation of MMP-9 transcription levels and production triggers macrophage invasion and inflammation of the arterial wall, which promotes inflammation within the atherosclerotic plaque and subsequent progression of AS formation (Borst et al., 2015).

### 2.2 mTOR signaling pathway-associated autophagy and AS

Autophagy is an evolutionarily conserved subcellular process that degrades damaged protein aggregates in organelles and the cytoplasm (Wen et al., 2021). According to the different methods of cargo delivery to the lysosomal lumen, autophagy is divided into macroautophagy, microautophagy, and chaperone-mediated autophagy. The autophagy referred to here is macroautophagy (Liu et al., 2022). Autophagy plays a protective role in AS by degrading proteins and necrotic organelles to ensure cellular health and homeostasis (Henderson et al., 2021; Xu et al., 2021). Defective autophagy exacerbates cholesterol crystal-mediated hyperactivation of macrophage inflammatory vesicles and their pro-atherogenic IL-1β response (Razani et al., 2012). Research evidence shows that inhibitors of the mTOR pathway enhance the stability of atherosclerotic plaque by inducing autophagy (Ma et al., 2016). Lipophagy is a special type of autophagy, a process in which lysosomes degrade lipids, release fatty acids, and convert them to ATP (Shin, 2020). Prior research suggests that lipophagy degraded cytosolic lipid droplets, reduced the accumulation of intracellular free fatty acids (FFAs) and lipotoxicity, and promoted cholesterol efflux in foam cells. However, the specific mechanism of lipophagy in AS needs to be further studied (Liu et al., 2020; Robichaud et al., 2021; Zheng et al., 2021).

The mTOR signaling pathway regulates the upstream of autophagy in multiple cell types, including macrophages, VECs, and VSMCs. mTORC1 is a crucial regulator of autophagy with a negative regulatory effect through both direct and indirect approaches. mTORC1 directly phosphorylates unc-51-like kinase 1 (ULK1) and autophagy-related protein 13 (ATG13) (Kim et al., 2011). ULK1 is a homologue of yeast ATG1 that forms as a complex with ATG13, which can tightly regulate autophagy (Hosokawa et al., 2009; Jung et al., 2009). Meanwhile, mTOR indirectly inhibits autophagy by blocking lysosomal biogenesis by inhibiting the nuclear translocation of transcription factor EB (TFEB) (Martina et al., 2012; Napolitano et al., 2018). Defective autophagy releases more TNF-α, exacerbating further endothelial cell damage and increasing the expression of intercellular adhesion molecule 1 (ICAM-1), aggravating AS (Vion et al., 2017). Additionally, p62 is known as one of the specific substrates degraded by the autophagy-lysosomal pathway and its elevated levels often indicate autophagia dysfunction (Lippai and Low, 2014). Microtubule-associated protein 1 light chain 3 (LC-3) is located on the surface of the autophagosome membrane and participates in the formation of autophagosome, and LC3-II is a conjugated form of LC3 protein (Mizushima and Yoshimori, 2007). P62 and LC3-II (LC3II/LC3I ratio) are considered as the binding components of the core autophagy machinery and typical biomarkers associated with autophagic flux (Mizushima et al., 2010). As previously published studies describe, intracellular expression of p62 and LC3-II was significantly up- and downregulated, respectively, at sites of AS and frequently served as biomarkers of autophagy levels (Wang et al., 2016b; Zhang et al., 2018; Pattarabanjird et al., 2022; Sabbieti et al., 2022). This phenotypic alteration was also observed in ox-LDL-inhibited VSMCs *via* inhibiting the PI3K/AKT/mTOR signaling pathway (Qin et al., 2022). Several studies have also shown that increasing autophagy by activating the upstream PI3K/AKT pathway and inhibiting the activity of the mtor signaling pathway can attenuate ox-LDL-induced endothelial cell injury (Zhang et al., 2021a; Zhou et al., 2022b). Research in Dr. Babak Razani's lab showed that in macrophages, a high protein diet induces the activation of the leucine-mediated mTOR signaling pathway; mitochondrial autophagy is inhibited, exacerbating mitochondrial dysfunction and macrophage apoptosis, accelerating the progression of AS (Zhang et al., 2020). Interestingly, silencing of the RICTOR, the major subunit of mTORC2, increased LC3-II expression and decreased p62 protein levels in endothelial cells, suggesting that mTORC2 can also interfere with autophagic flux by a yet-to-be-fully elucidated mechanism (Bernard et al., 2014).

### 2.3 mTOR signaling pathway-mediated cell senescence in the pathogenesis of AS

Cell senescence is a state of indefinite cell cycle arrest implemented in response to sublethal stresses (Hwang et al., 2022). Current studies have found that dietary restriction (DR) without malnutrition is the most effective way to prevent cell senescence in humans, and *in vivo* experiments revealed that inhibition of mTORC1 activity in yeast, nematodes, flies, and mammals can lead to a longer lifespan (Vellai et al., 2003; Kapahi et al., 2004; Kaeberlein et al., 2005; Wu et al., 2013; Green et al., 2022). Excessive nutrient and lipid stimulation, the triggers of cellular senescence, activate the signaling pathways upstream of the mTOR signaling pathway, including those of PI3K/AKT, AMPK, and Rag-GATOR, resulting in increased mTORC1 activity, and then regulating the downstream cellular activities, such as protein synthesis and autophagy (Bent et al., 2016; Hesketh et al., 2020; Sadria and Layton, 2021). Existing studies suggest that the involvement of the mTOR pathway is central to cell senescence, mainly for the properties of mTOR inhibitors (e.g., Sirolimus and everolimus) available in humans. However, they are not primarily aimed at aging and have many side effects (primarily insulin resistance and immunosuppression) (Cruzado et al., 2016; Carosi et al., 2022).

AS is an age-related disorder, and the incidence of AS increases significantly with age. Age-related cell senescence is a key element in the pathogenesis of AS (Bjorkegren and Lusis, 2022). On the one hand, cell senescence blocks the cell cycle to G_1_-G_2_ phase and inhibits damaged cell proliferation, which has a protective effect in certain cases of cancer (Huang et al., 2022b). On the other hand, cell senescence also leads to hyperfunction in cells; studies have shown that the secretion of inflammatory factors in senescent cells is increased, and such pathological changes will aggravate the progression of AS (Bian et al., 2020; Xiang et al., 2022). Large VECs similar to senescent cells *in vitro* are often found on the surface of plaques, suggesting that vascular cells may undergo senescence *in vivo* (Minamino et al., 2002). Research has shown increased mTOR activity in senescent VSMCs induced by Adriamycin, and inhibiting the mTOR signaling pathway significantly decreased the expression of senescence markers (p53/p21/p16) (Sung et al., 2018). In addition, a decrease in the level of telomeric repeat-binding factor 2 (TRF2) in VSMCs derived from human plaques suggests the emergence of cell senescence and promotes endothelial dysfunction (Wang et al., 2015). Remarkably, in a murine model of AS, Rapamycin was used to enhance autophagy, and the mTORC1/ULK1/ATG13 signaling pathway was found to delay the senescence of mouse smooth muscle cells (Luo et al., 2017). Meanwhile, this study also revealed that inactivation of mTORC1 could inhibit p53 to suppress VSMC senescence. Subsequent studies elaborated upon the possible role of mTOR-p53-p21 signal cascades in cell senescence (Brooks and Gu, 2010; Huang et al., 2022b). However, whether this mechanism functions in AS, remains to be investigated.

### 2.4 Regulation of mTOR signaling pathway in AS lipid metabolism

It is well known that dyslipidemia is closely related to the occurrence and development of AS (Authors/Task Force et al., 2019). mTORC1 promotes fat storage by inhibiting lipolysis and promoting *de novo* lipogenesis, which may be related to the downstream of mTORC1/S6K signaling factors like sterol-regulatory element binding proteins (SREBPs), an important family of transcription factors that promotes lipogenesis and adipogenesis, and control lipid metabolism (Chakrabarti et al., 2010; Cheon and Cho, 2021). mTORC1 has been shown to regulate SREBP by controlling the nuclear import of phosphatidic acid phosphatase lipin 1 (Libby, 2021b). *In vivo* experiments reversed the inflammation-induced LDLr increase in ApoE^−/−^ mice by the Rapamycin inhibitor and verified that increased mTORC1 activity upregulates SREBP-2-mediated cholesterol uptake through retinoblastoma tumor suppressor protein phosphorylation (Ma et al., 2013). By blocking mTOR expression using specific small interfering RNA (siRNA), it was observed that macrophage-derived foam cell formation was inhibited, accompanied by a reduction in lipid deposition (Wang et al., 2014). Meanwhile, mTORC1 can regulate lipolysis and adipose tissue thermogenesis by regulating the downstream GRB10 (Liu et al., 2014). Nevertheless, treatment of adipocytes with Rapamycin reduced insulin-stimulated TAG stores by about 50%, and the same trend was observed in RAPTOR knockout mice, which showed an increase in serum-free fatty acids, one of the reasons why mTOR inhibitor use often results in high triglyceride lipids (Soliman et al., 2010; Paolella et al., 2020). Hypertriglyceridemia is also a significant cause of AS, which has recently attracted more and more attention (Libby, 2021b). Therefore, modulation of mTORC1 activity in lipid regulation of AS is controversial, although most studies have demonstrated a significant inhibitory effect of mTOR inhibitors on plaque growth. Beyond this, it has also been shown that adipocyte-specific mTORC2 activity promotes adipogenesis, *de novo* lipogenesis, and controls insulin-stimulated glucose uptake, but direct evidence for these processes associated with AS is lacking (Tang et al., 2016; Szwed et al., 2021).

## 3 Natural compounds and anti-atherosclerosis

**FIGURE 4:**
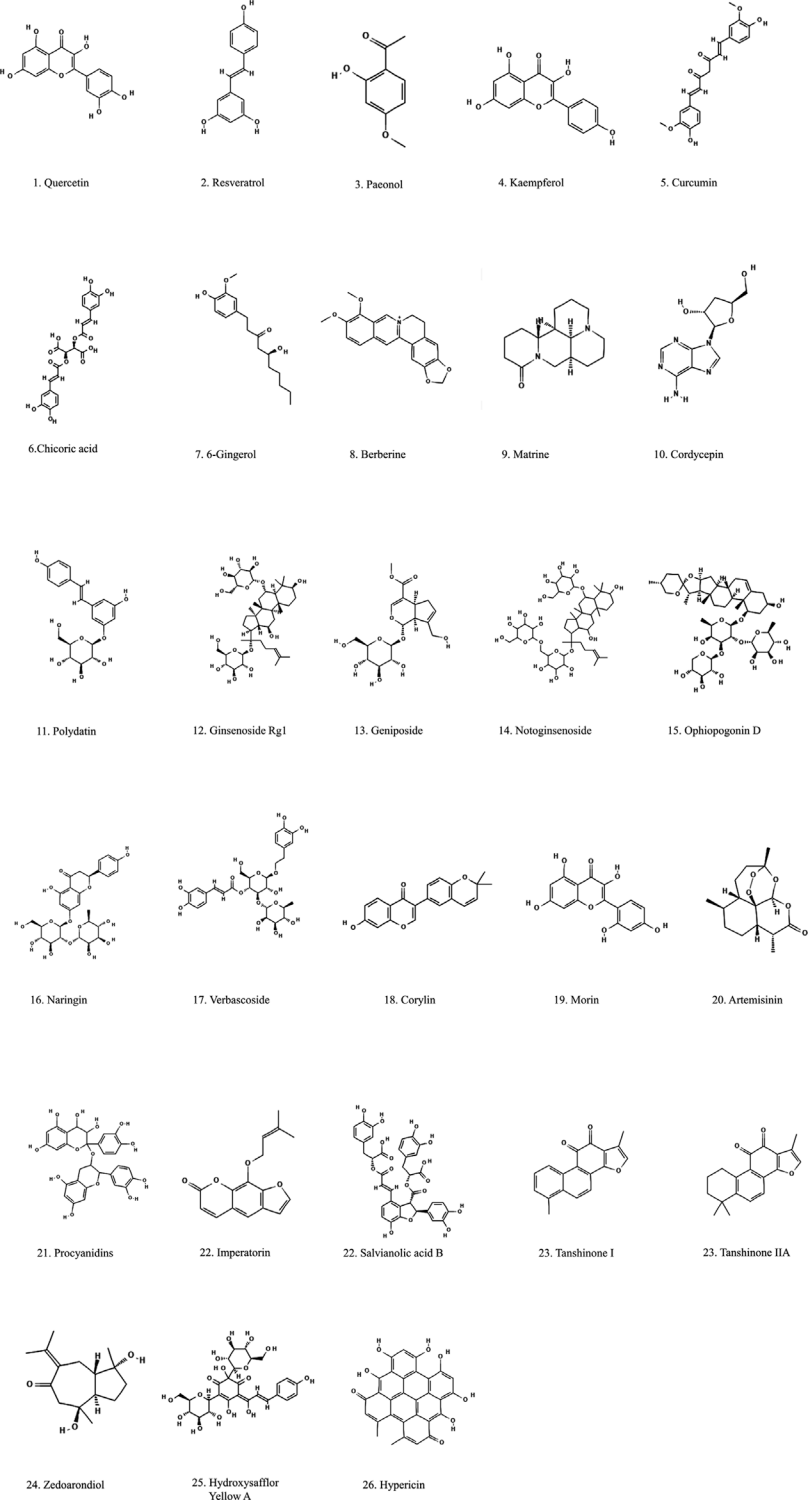
Structural formulation of natural compounds derived from botanical drugs with anti-atherogenic effects.

Many natural compounds extracted from botanical drugs have positive therapeutic effects on the occurrence and development of AS through various ways, such as promoting autophagy, regulating blood lipids, reducing inflammatory factors, alleviate cellular senescence, etc. (Xu et al., 2015; Rastogi et al., 2016; Lyu et al., 2022). In this paper, various natural compounds are divided into the following categories according to their chemical formulas: polyphenols, alkaloids, glycosides, and others, which are listed in Tables 1–Tables 4. The chemical composition is shown in Figure 4.

**TABLE 1:** Summary of the effects of polyphenolic natural compounds on different models of AS.

Active ingredients	Source	Experimental model	Dose/concentration	Efficiency	Molecular targets	Signaling pathway	References
Quercetin	*Cuscuta chinensis Lam.; Morus alba L.*	vivo: HFD fed 8weeks male ApoE^−/−^ mice; vitro: ox-LDL induced HAECs	vivo: 20mg/kg/d vitro: 3,1,0.3 mmol/L	Alleviate lipid deposition and atheroscle-rotic; alleviate cell senescence	Sirtuin↑ sIcam-1↓ IL-6↓ VCAM-1 ↓	//	Jiang et al. (2020)
Quercetin	*//*	HFD fed 12 weeks male ApoE−/− mice	12.5 mg/kg/d	Regulate blood lipid; Reduce inflammation; Induce autophagy	LC3 II/I↑	mTOR	Cao et al. (2019)
					p53↓		
					p21↓		
Resveratrol	*Morus alba L., Smilax glabra Roxb.*	6 weeks HFD fed male ApoE^−/−^ mice	50 mg/kg/day	Regulate blood lipid;	MMP-9↓	PI3K/AKT/mTOR	Ji et al. (2022)
				Reduce inflammation	CD40L↓		
Resveratrol	//	Ox-LDL induced rabbit SMC	25 μM	Inhibit cell proliferation	//	PI3K/AKT/mTOR/p70S6K	Brito et al. (2009)
Paeonol	*Paeonia × suffruticosa Andrews*	**Vivo:** 6 weeks male ApoE^−/−^ mice fed by a high-cholesterol diet **vitro:** ox-LDL treated VSMCs	**Vivo:** 400, 200, and 100 mg/kg/d **vitro:** 15,30,60 μM	Inhibit cell proliferation; Induce autophagy	α-SMA↓	AMPK/mTOR	Wu et al. (2017)
					LC3II↑ p62↓		
					LC3II/actin↑		
Kaempferol	*Ardisia japonica (Thunb.) Blume*	HUVECs induced by ox-LDL	50,100,200 μM	Attenuate cell injury; Induce autophagy	LC3-II/I↑ p-mTOR↓	AMPK/mTOR/p70S6K	Che et al. (2017)
Curcumin	*Curcuma longa L.*	H_2_O_2_ induced EA.hy926 cell line	5,20 μM	Induce autophagy;	p-AKT↓	AKT/mTOR	Guo et al. (2016)
				Reduce oxidative stress	p-mTOR↓		
					LC3-II↑		
Curcumin	*//*	Ox-LDL-induced HUVECs	5 μM	Regulate blood lipid; Reduce inflammation; Reduce oxidative; stress; Induce autophagy	LC3-II↑	AMPK/mTOR/p70S6K	Zhao et al. (2021)
Nicotinate-Curcumin	*//*	Ox-LDL-induced THP-1 cell line	10 μM	Induce autophagy	LC3-II↑ p62↓	PI3K-AKT-mTOR	Gu et al. (2016)
Hydroxyl Acetylated Curcumin (Sonodynamic therapy)	*//*	Human THP-1 monocytes	5.0 μg/mL	Reduce lipid accumulation; Induce autophagy	Beclin1↑ LC3-II↑ p62↓	PI3K/AKT/mTOR	Zheng et al. (2016)
Chicoric acid	*Cichorium intybus L.*	Vivo: Sprague-Daw-ley rats with ligated left common carotid artery; vitro: PDGF-BB induced VSMCs	**vivo:** 50 mg/kg/d **vitro:** 10,50,100 μM	Inhibit cell proliferation and migration	p-mTOR↓ PCNA↓	mTOR/P70S6K	Lu et al. (2018)
					cyclin D1↓		
					p27↓		
6-Gingerol	*Zingiber officinale Roscoe*	Hydrogen- peroxide induced HUVECs	10, 20, 40 μM	Reduce oxidative stress; Induce autophagy	LC3-II↑	PI3K/AKT/mTOR	Wang et al. (2016a)
					Bcl-2↑		
					Beclin1↑ p-AKT↓		
					p-mTOR↓		

↑, upgrade; ↓, downgrade.

**TABLE 2:** Summary of effects of natural alkaloids on different AS models.

Active ingredients	Source	Experimental model	Dose/concentration	Efficiency	Molecular targets	Signaling pathway	References
Berberine	*Coptis chinensis Franch.*	HFD fed 8-week-old male ApoE^−/−^ mice	78,117,156 mg/kg	Regulate blood lipid; Induce autophagy	Beclin-1↓ p62↓	PI3K/AKT/mTOR	Song and Chen, (2021)
					p-PI3K↑		
					p-mTOR↑		
					p-AKT↓		
Berberine	*//*	Murine cell line J774A incubated with ox-LDL	25,50 μM	Induce autophagy; Reduce inflammation	LC3 II/I↑ SQSTM1/p62↓	AMPK/mTOR	Fan et al. (2015)
Berberine (sonodynamic therapy)	*//*	Human THP-1 monocytes	30 μg/mL	Induce autophagy; Regulate cholesterol efflux	LC3-II/I↑ p62↓	PI3K/AKT/mTOR	Kou et al. (2017)
Matrine	*Sophora flavescens Aiton*	AGE- induced HCSMCs	0,0.25,0.5,0.75 and 1.0mmol/L	Suppress fibrotic response	PI3K↓	PI3K/AKT/mTOR/p70S6k	Ma et al. (2019)

↑, upgrade; ↓, downgrade.

**TABLE 3:** Summary of the effects of natural compounds of glycosides on different AS models.

Active ingredients	Source	Experimental model	Dose/concentration	Efficiency	Molecular targets	Signaling pathway	References
Polydatin	*Reynoutria japonica Houtt.*	HFD fed 8-week-old male ApoE^−/−^ mice	50 mg/kg	Induce autophagy	LC3-Ⅰ/II↑ p62↓	PI3K/AKT/mTOR	Xiong et al. (2021)
Ginsenoside Rg1	*Panax ginseng C.A.Mey.*	Murine Raw264.7 macrophages	20,50,100,200 μM	Induce autophagy	Bcl-2↑	AMPK/mTOR	Yang et al. (2018)
					Bax↓		
					LC3↑ p62/SQSMT1↑		
Geniposide combined with Notoginse-noside	*Gardenia jasminoides J.Ellis* and *Panax notoginseng (Burkill) F.H.Chen*	vivo: HFD fed 8 weeks-old male ApoE^−/−^ mice vitro: H2O2 induced HUVECs	vivo: Geniposide 50 mg kg^−1^ + Notoginsenosid50 mg kg^−1^ vitro: Geniposide100 μM + Notoginseno-sid 100 μM	Alleviate cells damage; Induce autophagy; Reduce inflammation	NLRP3↑	AMPK/mTOR/Nrf2	Liu et al. (2021)
					caspase-1↑		
					VCAM-1↑		
Ophiopogonin D	*Ophiopogon japonicus (Thunb.) Ker Gawl.*	vivo: HFD fed 8-week-old male ApoE^−/−^ mice vitro: LO_2_ cells	vivo: 0.5 mg/kg/d vitro: 20,40,80 mM	Regulate blood lipid	p-mTOR↓	mTOR/SREBP1/SCD1	Zhang et al. (2021b)
					SREBP1↓		
					SCD1↓		
Naringin	*Citrus × aurantium L.;*	TNF-α-induced VSMCs	10,15,25 mM	Inhibit cell invasion and migra-tion	MMP-9↓	PI3K/AKT/mTOR/p70S6K	Lee et al. (2009)
	*Plantago lanceolata L.*				NF-κB↓		
Verbascoside	*Plantago lanceolata L.;*	HFD fed male wistar rats	2 mg/kg	Regulate blood lipid; Reduce inflammation	MMP-9↓	AMPK/mTOR	Fan and Zhang, (2021)
	*Forsythia suspensa (Thunb.) Vahl*						

↑, upgrade; ↓, downgrade.

**TABLE 4:** Summary of the effects of other natural compounds on different AS models.

Active ingredients	Source	Experimental model	Dose/concentration	Efficiency	Molecular targets	Signaling pathway	References
Corylin	*Cullen corylifolium (L.) Medik.*	PDGF-BB induced VSMCs	20 μ M	Inhibite proliferation and migration in VSMCs	CDK4↓	mTOR/Drp1	Chen et al. (2020)
					CDK2↓		
					Cyclin D1↓		
					Cyclin E↓		
Morin	*Morus alba L.;*	Ox-LDL induced HUVECs	1,3,10,30 μmol/L	Attenuate cell injury; Induce autophagy	LC3↓ p62↑	AMPK/mTOR	Zhang et al. (2019)
					p-AMPK↑		
	*Prunella vulgaris L.*				p-mTOR↓		
Artemisinin	*Artemisia caruifolia var. caruifolia*	HFD fed 8weeks male ApoE^−/−^ mice	50,100 mg/kg/d	Reduce inflammation; Induce autophagy	p62↓	AMPK/mTOR/ULK1	Cao et al. (2020)
					LC-3II↑		
Artemisinin and Procyanidins loaded multifunctional nanocomplexes	*Artemisia annua L.; Forsythia suspensa (Thunb.) Vahl*	vivo: HFD fed 6-week-old male ApoE^−/−^ mice vitro: RAW264.7 stimulation with lipopolysaccharide	vivo: 0.9 mg/kg procyanidins +4.1 mg/kg artemisinin vitro: RAW264.7 stimulation with lipopolysaccharide	Inhibiting the formation of atheroscler-otic plaques; inhibitory inflammatory cytokines; romote cholesterol efflux and lipid influx	AMPK ↑	NF-κB/NLRP3; AMPK/mTOR	Zhou et al. (2022a)
					Beclin-1 ↑		
					LC3II/I↑		
					SR-BI↑		
					ABCA-1 ↑		
					ABCG-1 ↑		
Imperatorin	*Angelica dahurica (Hoffm.) Benth. & Hook.f. ex Franch. & Sav.*	Ox-LDL induced VSMCs	10,20,40 μM/L	Attenuate VSMCs migration, alleviate foam cell formation	p-PI3k↓	PI3K/AKT/mTOR	Li et al. (2020)
					p-AKT↓		
					p-mTOR↓		
Salvianolic acid B	*Salvia miltiorrhiza Bunge*	Cholesterol crystal-induced RAW264.7 cell line	100,150,200 µM	Attenuate cholesterol crystals; improve the autophagy	LC3-II↑ Beclin-1↑ p62↓	AKT/mTOR	Sun et al. (2021)
Salvianolic acid B	*//*	RAW264.7 cells	100,150,200 µM	Promote autophagy	p-AKT↓ p-mTOR↓	NF-κB/AKT/mTOR	Zou et al. (2022)
Salvianolic acid B	*//*	Hydrogen peroxide- induced HUVECs	5,10,20 μ g/ml	Promote autophagy	caspase-3↓	AMPK/mTOR	Gao et al. (2019)
					LC3-Ⅱ↑		
					Beclin-1↑ p62↓		
Zedoarondiol	*Curcuma aromatica Salisb.*	PDGF-BB induced VSMCs	5,10,20 μg/mL	Inhibit VSMCs proliferation	p53↑	AMPK/mTOR/p70S6K	Mao et al. (2016)
					p21↑		
Hydroxysafflor Yellow A (Sonodynamic Therapy)	*Carthamus tinctorius L.*	Human THP-1 monocytes	0.1,0.3,0.6,0.8,1mmol/L	Induce autophagy, inhibite inflammatory factors	LC3-II↓	PI3K/AKT/mTOR	Jiang et al. (2017)
					Beclin↓		
					p62↓		
Hypericin-mediated (sonodynamic Therapy)	*Sedum sarmentosum Bunge*	Human THP-1 monocytes	0.25 μg/mL	Activate autophagy	LC3-II/I↑	AMPK/AKT/mTOR	Li et al. (2016)
					Beclin↑		
					SQSTM1/p62↓ p-AMPK↑		

↑, upgrade; ↓, downgrade.

### 3.1 Polyphenols

As a kind of secondary metabolite, polyphenols are widely found in a variety of botanical drugs. Existing research on polyphenols mainly focuses on their anti-inflammatory, antioxidant, and anticancer effects (Cheng et al., 2017; Benvenuto et al., 2020). It is worth noting that some polyphenols, including resveratrol, paeonol, and curcumin, may display anti-AS properties by interacting with some cell receptors and/or gene expression regulators through mTOR and its related effectors due to their unique biological activity(Lu et al., 2018; Tian et al., 2019).

Quercetin is a flavonoid compound widely found in various vegetables, fruits, berries, and in many botanical drugs such as *Cuscuta chinensis Lam.* and *Morus alba L.* It has a wide range of biological activities, including anti-inflammatory, anti-aging, antioxidant, etc. (Stewart et al., 2008; Kobori et al., 2016; Cui et al., 2022). Quercetin significantly reduced atherosclerotic plaque area, aortic lipid accumulation and serum inflammatory factor levels in mice (Sun et al., 2015; Ren et al., 2018; Jia et al., 2019). It has been observed that quercetin inhibits the development of atherosclerotic plaques in mice by activating the PI3K/AKT signaling pathway, which is also upstream of the mTOR signaling pathway (Lu et al., 2017). Also, quercetin could enhance autophagic flux and reduce lipid accumulation in the aortic roots of hfd-fed mice by inhibiting mTOR expression (Cao et al., 2019). Jiang et al. (2020) found that quercetin (20 mg/kg/d) gavage for 8 weeks could significantly inhibit AS in mice as well as increase the density of Sirt 1 in mice aorta, the changes of which are related to the decline of autophagy in the cell senescence. Although this study concluded by KEGG analysis stated that the mTOR signaling pathway is involved in the pharmacological mechanism of quercetin against ox-LDL, a rigorous experimental validation is lacking. Notably, some studies suggest that flavonoids represented by quercetin are prone to aggregation behavior and non-specific inhibition, which raises questions about the reliability of reports on the biological activity of polyphenols *in vitro* (Pohjala and Tammela, 2012; Sheridan and Spelman, 2022). It is also interesting to note that quercetin has been reported to have low solubility and poor bioavailability, which limits its applicability (Abdel-Tawab, 2021; Bertelli et al., 2021). Therefore, how to improve the bioavailability of these valuable compounds is also a promising topic to be explored in current and future studies (Sapino et al., 2015).

Resveratrol, a typical polyphenol compound, can be obtained from *Morus alba L*, *Smilax glabra Roxb.,* and other botanical drugs. Resveratrol has a variety of biological activities, and has been reported to protect the permeability of endothelial cells and maintain the integrity of the endothelial barrier, which has a protective effect against AS (Gurusinghe et al., 2017). Early studies found that the mTOR signaling pathway is activated by ox-LDL and is associated with VSMC proliferation; resveratrol inhibits DNA synthesis and proliferation of rabbit VSMCs by blocking ox-LDL-induced phosphorylation and activation of the PI3K/AKT/mTOR/p70S6K pathway (Brito et al., 2009). Ji et al. (2022) investigated the relationship between resveratrol and mTOR in umbilical vein endothelial cells (UVECs) in AS mice models. After intraperitoneal injections of resveratrol (50 mg/kg/day) for 5 weeks, resveratrol treatment significantly reduced the mRNA and protein expression levels of PI3K, AKT, and mTOR in UVEC, decreased serum inflammatory cytokine levels, and reduced the area of atherosclerotic lesions, but the relationship between these processes has not been demonstrated in the study.

Paeonol is the main active ingredient of *Paeonia × suffruticosa Andrews*, which has shown good anti-atherosclerotic effects in *in vitro* and *in vivo* experiments. Danserol could delay AS through processes such as inhibiting inflammation, regulating autophagy, promoting cholesterol efflux to inhibit foam cell production, and protecting vascular endothelial cells, which are processes closely related to the mTOR signaling pathway (Li et al., 2009; Li et al., 2015; Li et al., 2018; Liu et al., 2018). An investigation showed that high doses of salvianol (400 mg/Kg) had a good protective effect in mice with AS models. However, free drug plasma concentrations were not measured in this study to better understand the metabolism of the drug *in vivo*. Meanwhile, VSMC were treated *in vitro* using 30 μM dermatol, and the results showed that dermatol could activate autophagy and inhibit the proliferation of vascular smooth muscle cells by activating the AMPK/mTOR signaling pathway (Wu et al., 2017). In addition, research has shown that paeonol acts against premature senescence by modulating Sirtuin 1 pathway (Jamal et al., 2014). Although other studies have linked mTOR with Sirtuin 1 in the process of autophagy and senescence (Morselli et al., 2010; Liu et al., 2016), whether paeonol can regulate senescence through mTOR signaling still needs to be investigated.

Curcumin is a natural polyphenol, the most active ingredient of *Curcuma longa L*, which may be a potential multi-target therapeutic avenue for atherosclerotic diseases. Guo et al. (2016) conducted an *in vitro* experiment and found that compared to blank group and H_2_O_2_-treated (200 µmol/L) group, the expression level of LC3-Ⅱ protein in curcumin-pretreated (5, 20 µmol/L) group was significantly increased, while the expression levels of p-AKT and p-mTOR were significantly decreased, suggesting AKT’s involvement in curcumin-induced mTOR inhibition and autophagy to protect endothelial cells. Similarly, curcumin plays an important role in attenuating atherosclerotic endothelial cell injury by regulating the autophagy-related PI3K/AMPK/mTOR/p70S6K signaling pathway (Zhao et al., 2021). Curcumin promotes autophagy and reduces inflammation by promoting nuclear translocation in TFEB, one of the downstream factors of mTORC1 (Li et al., 2022). However, whether curcumin can regulate TFEB through mTOR still needs more specific studies. In addition, the current studies are using *in vitro* experiments to probe the pharmacological activity of curcumin targeting the mTOR signaling pathway, which is not always representative of the natural physiological environment and therefore may not provide an accurate prediction for *in vivo* botanical drugs.

Chicoric acid is a potent anti-atherosclerotic component that has been shown to have strong cardiovascular protective effects. Studies have shown that it alleviates endothelial dysfunction by attenuating ox-LDL (Tsai et al., 2017). Lu et al. (2018) conducted experiments to investigate the potential mechanism of chicoric acid in treating AS. They ligated the left carotid artery of rats for 8 weeks and found that 8 weeks of chicoric acid (50 mg/kg/d) gavage significantly inhibited carotid intimal hyperplasia and reduced the intima area of the injured carotid artery; western blot analysis showed that chrysotile acid decreased p-mTOR and p-P70S6K protein levels, suggesting its possible inhibition of the mTOR/P70S6K signaling pathway, which in turn hindered the VSMC phenotypic switching, proliferation, migration, and neointima formation and improving AS.

6-Gingerol is an important active component of *Zingiber officinale Roscoe* with anti-inflammatory and antioxidant abilities (Ballester et al., 2022). 6-gingerol reversed the significant reduction in oxidative stress-induced levels of LC3-II, Beclin1 (key protein for upregulation of autophagy), and Bcl-2 (key gene for inhibition of apoptosis) in HUVECs and decreased the expression of p-AKT and p-mTOR, demonstrating that 6-gingerol can induce autophagy through the AKT/mTOR pathway, avoid apoptosis, and protect vascular endothelial cell survival (Wang et al., 2016a). This experiment also provided research insights for further investigation of the possible mechanisms of the interaction between mTOR-mediated autophagy and apoptosis.

### 3.2 Alkaloids

Alkaloids are also a type of secondary metabolites, which are mainly derived from plants, but are also found in animals and fungi. Emerging evidence suggests that alkaloids have a wide range of biological activities (Mondal et al., 2019; Shang et al., 2020). Its mechanism includes the inhibition of cell proliferation, regulation of autophagy, and regulation of a variety of related genes and pathways. Therefore, the potential of alkaloids to inhibit AS is worth investigating.

Berberine, an isoquinoline alkaloid, widely exists in *Coptis chinensis Franch*. A recent study reported the potential mechanism of berberine in treating AS (Song and Chen, 2021). High-fat diet (HFD)-fed ApoE^−/−^ mice were divided into a model group, positive drug group, and low (78 mg/kg), medium (117 mg/kg), and high dose (156 mg/kg) berberine groups. Interestingly, compared to the model group, all doses of berberine reduced AS plaque area and serum TC, TG, and LDL-C levels, while only high doseof berberine had an effect on HDL-C levels; middle and high doses had effects on the expressions of p-PI3K, p-mTOR, and p-AKT, suggesting that the biological effects of berberine in treating AS may be through the PI3K/AKT/mTOR signaling pathway. Another report (Fan et al., 2015) showed that berberine reduced ox-LDL-induced inflammation in a dose-and time-dependent manner and increased LC3II/LC3I and SQSTM1/p62 levels, indicating that berberine inhibited inflammation by upregulating autophagy; the activation of AMPK/mTOR signaling pathway stimulates autophagy in macrophages, and chloroquine could reduce these effects. Induction of ROS production and autophagy are important in regulating macrophage lipid deposition through lysosome/autophagosome-mediated degradation. Berberine-mediated sonodynamic therapy may become a new treatment option for AS, which promotes ROS production, induces cholesterol efflux, and induces autophagy in macrophages and foam cells through PI3K/AKT/mTOR signaling pathway inhibition (Kou et al., 2017).

Matrine is a tetracyclo-quinolizindine alkaloid, which can be extracted from *Sophora flavescens Aiton*. Advanced glycosylation end products (AGEs) are a group of substances existing in poorly controlled type 2 diabetes mellitus, which promote the fibrotic response of VSMCs and then aggravate AS through mTOR signaling pathway activation (de Vos et al., 2016). Matrine pretreatment reduced the expression of PI3K and the phosphorylation of mTOR in a concentration-dependent manner, as well as inhibited AGEs-induced fibrosis response in human coronary smooth muscle cells (HCSMCs) (Ma et al., 2019). The regulatory effect of matrine on mTOR provides a new idea for the treatment of type 2 diabetes-associated AS.

### 3.3 Glycosides

Glycosides are organic molecules whose therapeutic activities are manifested in many aspects. Some glycosides such as Polydatin, Astragaloside IV, and Ginsenoside Rg1, showed excellent anti-AS effects.

Polydatin has a wide range of pharmacological activities, and is a natural component extracted from *Reynoutria japonica Houtt.* Xiong and his colleagues (Xiong et al., 2021) observed high expression levels of PI3K (p-PI3K), AKT (p-AKT) and mTOR (p-mTOR) proteins in atherosclerotic plaques of ApoE^−/−^ mice induced by a HFD. Polydatin intervention significantly reduced this effect and was accompanied by an elevated autophagic flux. The addition of 3-Methyladenine (3-MA) in the study inhibited autophagy and reversed the downregulation of p-mTOR by polydatin, suggesting that the mechanism of polydatin-mediated AS inhibition in the *in vivo* experiments may be by the regulation of autophagy through the mTOR signaling pathway.

Ginsenoside Rg1 is derived from *Panax ginseng* C.A. Mey and has cardiovascular protective and anti-inflammatory effects (Gao et al., 2020; Sarhene et al., 2021). Ginsenoside Rg1 ameliorates ox-LDL-induced apoptosis, senescence, and oxidative stress in HUVECs cells through the AMPK/SIRT3/p53 signaling pathway (Lyu et al., 2022). More experiments are needed to prove whether mTOR is involved in the anti-senescence effect of ginsenoside Rg1. Another study reported that ginsenoside Rg1, at the most effective dose of 50 uM, could inhibit ox-LDL-induced apoptosis of Raw264.7 macrophages by activating the AMPK/mTOR signaling pathway to upregulate autophagic flux, which is a protective factor in advanced AS, as excessive macrophage apoptosis increases atherosclerotic plaque instability (Yang et al., 2018). However, the relationship between mTOR-mediated autophagy and apoptosis in AS still requires new experimental evidence, as some studies also consider that autophagy and apoptosis are two different types of processes (Shan et al., 2021).

Ophiopogonin D is an effective compound isolated from botanical drugs *Ophiopogon japonicus (Thunb.) Ker Gawl.* Ophiopogonin D treatment can reduce blood lipid levels and alleviate mitochondrial damage and dysfunction in palmitic acid–stimulated mice(Li et al., 2021). Gavage of HDF-fed mice with Daidzein D (0.5 mg/kg/D) for 12 weeks significantly reduced lipid levels, mTOR and p-mTOR levels, SREBP1 and SCD1 levels, and aortic root plaques, suggesting that Daidzein D may decrease lipogenesis and alleviate AS by inhibiting phosphorylation of mTOR, and thus SREBP1 and SCD1 (Zhang et al., 2021b).

### 3.4 Others

Artemisinin is an endoperoxide sesquiterpene lactone mainly derived from *Artemisia caruifolia var. caruifolia*, which can be used as a potential therapeutic agent for AS. Cao et al. (2020) reported that artemisinin’s (50,100 mg/kg) intragastolic administration for 8 weeks effectively reduced the formation of AS plaque in ApoE^−/−^ mice and downregulated the levels of inflammatory factors such as MCP-1, IFN-γ, IL-6, and TNF-α. In addition, *in vitro* experiments showed that artemisinin (100 μM) promoted the autophagy of macrophages after ox-LDL treatment and inhibited the production of inflammatory cytokines, along with the upregulation of AMPK activation and the inhibition of phosphorylation of mTOR and ULK1. Another report showed that artemisinin and Procyanidins co-loaded nanocomplex can inhibit the RONS/NF-κB/NLRP3 pathway to reduce inflammation and inhibit lipid influx, and enhance the AMPK/mTOR pathway to regulate cholesterol efflux, thereby regulating lipid metabolism to attenuate atherosclerotic lesions (Zhou et al., 2022a).

Salvianolic acid B (Sal B) is a flavonoid compound, which is isolated from the botanical drug *Salvia miltiorrhiza Bunge*, it can inhibit the development of arterial plaque in ApoE^−/−^ mice, reduce the production of ox-LDL in serum, and show significant anti-inflammatory effects (Yang et al., 2020). Sal B (200 μM) inhibited the LPS + IFN-γ-activated AKT/mTOR pathway and enhanced autophagy, while insulin activation of the mTOR signaling pathway counteracted the inhibitory effect of Sal B on M1 macrophage polarization, suggesting that Sal B can inhibit M1 macrophage polarization and reduce the release of inflammatory factors through the mTOR signaling pathway, thus exerting an anti-atherogenic effect (Zou et al., 2022).

Tanshinone I and Tanshinone IIA are also important active substances extracted from *Salvia miltiorrhiza Bunge.* According to a recent report, tanshinone I significantly inhibited the activation of PI3K and the phosphorylation of mTOR, 70S6K, and S6 in a concentration-dependent manner, thereby inhibiting the proliferation of VSMCs and playing a protective role in AS (Wang et al., 2020b). Tanshinone IIA can phosphorylate endothelial oxide synthase (eNOS) by activating TGF-β/PI3K/AKT pathway, promote the product and release of endogenous nitric oxide, and protect vascular endothelial cells (Wang et al., 2020a). Tanshinone IIA can also down-regulate the activity of CD40 and MMP-2, downstream factors of mTOR, in HFD-induced AS rabbits for anti-inflammation, but whether this pathway is through mTOR has not been confirmed (Fang et al., 2008).

## 4 Conclusion and perspectives

The natural compounds derived from botanical drugs containing quercetin, resveratrol, and other botanical drugs have the characteristics of multi-target, multi-pathway collaboration, by which they perform anti-atherosclerotic effects through lipid-lowering, anti-inflammatory, slowing down the cell senescence, and autophagy-promoting mechanisms (Zhi et al., 2023). The mTOR signaling pathway plays an important role in the formation and development of AS. We summarized the effects of the mTOR signaling pathway from the immune response, autophagy, anti-aging, and regulation of lipid metabolism in atherogenesis, suggesting that mTOR signaling pathway inhibition may be a potential therapeutic target for AS. In current clinical studies, mTOR inhibitors are not applicable to the primary treatment of AS since they target the activity of the mTOR complex, which has a great impact on the cell cycle and causes disturbances in glucose and lipid metabolism, instead of being one of the risk factors for AS (Wang et al., 2022). On the contrary, numerous studies have shown that natural botanical drugs can effectively reduce lipid levels, while also inhibiting the mTOR signaling pathway, elevating autophagy and reducing immune response, indicating the advantages of TCM (Song et al., 2019; Zhou et al., 2022a).

Although a large body of preclinical evidence implicates the mTOR signaling pathway in the regulation of AS, there remain many limitations in the study of the effects and mechanisms of natural compounds that modulate the mTOR pathway in treating AS. The multi-targeted actions of TCM drugs may trigger additional effects upstream and downstream of the mTOR signaling pathway, generating some impacts other than anti-AS that were not all detectable throughout the experiments. Many studies have broadly attributed the regulatory effects of drugs on the mTOR signaling pathway to the regulation of protein synthesis by mTORC1. While this can be verified experimentally, whether it interferes with protein expression through other more complex molecular biological processes remains to be discovered. In the recent experimental studies on TCM, few have detected the expression levels of proteins in the mTORC1 and mTORC2 complexes other than the mTOR proteins, as well as these proteins playing a crucial role in the mTOR signaling pathway. In addition, existing studies are limited to the scope of animal or cellular experiments, and evidence based on the results of high-quality clinical studies is lacking, possibly due to the difficulty of extracting natural products from botanical drugs and their low bioavailability. The side effects of phytomedicines also need to be clarified before their possible clinical application. These limitations suggest additional directions for future fundamental research and clinical trials.

In conclusion, we have reviewed that the natural products of botanical drugs can slow down the progression of AS by inhibiting the mTOR signaling pathway. This work provides a theoretical basis and a reference for future research to further expand the mechanisms and applications of natural products of TCM. However, additional studies are needed to elucidate the specific mechanisms involved in order to achieve the ultimate goal of botanical drugs used in TCM for the prevention and treatment of AS.

## Glossary

3-MA: 3-Methyladenine
4E-BP1: 4E-binding proteins 1
AGE: advanced glycation end product
AS: Atherosclerosis
ATG13: autophagy-related protein 13
CVD: cardiovascular disease
CRP: C-reactive protein
DR: dietary restriction
eNOS: endothelial oxide synthase
FFA: fatty acids
HDL-C: high-density lipoprotein cholesterol
HFD: high-fat diet
HCSMCs: human coronary smooth muscle cells
HUVECs: human umbilical vein endothelial cells
LC-3: microtubule-associated protein 1 light chain 3
LDL-C: low density lipoprotein cholesterol
ICAM-1: intercellular adhesion molecule 1
IFN-γ: Interferon-γ
IL-1β: interleukin-1β
IL-6: interleukin 6
LDLR−/−: low-density lipoprotein receptor knockout
mTOR: mechanistic/mammalian target of rapamycin
mTORC1: mammalian target of rapamycin complex 1
MCP-1: monocyte chemoattractant protein-1
mTORC2: mammalian target of rapamycin complex 2
NF-κB: nuclear factor-κB
ox-LDL: oxidized low-density lipoprotein
p70S6K: 70-kDa ribosomal protein S6 kinase
PBS: phosphate buffer saline
PI3K: phosphatidyl inositol 3-kinase
PIKKs: phosphatidyl inositol 3-kinase related kinases
PKC: protein kinases C
Raptor: regulatory protein associated with mTOR
Rheb: Ras homolog enriched in the brain
Rictor: rapamycin-insensitive companion of mTOR
S6K: S6 kinase
Sal B: Salvianolic acid B
SCD1: stearoyl-CoA desaturase-1
SGK1: serum-and glucocorticoid-induced protein kinase 1
sin1: serum-and glucocorticoid-induced protein kinase 1
siRNA: small interfering RNA
SMCs: smooth muscle cells
SREBPs: sterol-regulatory element binding proteins
TC: total cholesterol
TG: triglyceride
TCM: Traditional Chinese Medicine
TFEB: transcription factor EB
TLR-4: Toll-like receptor-4
TNF-α: tumor necrosis factor-alpha
TRF2: telomeric repeat-binding factor 2
TSC1/2: tuberous sclerosis complex 1/2
ULK1: unc-51-like kinase 1
UVECs: umbilical vein endothelial cells
VSMCs: vascular smooth muscle cells
VECs: vascular endothelial cells
